# Mitochondrial Diabetes in Children: Seek and You Will Find It

**DOI:** 10.1371/journal.pone.0034956

**Published:** 2012-04-19

**Authors:** Cristina Mazzaccara, Dario Iafusco, Rosario Liguori, Maddalena Ferrigno, Alfonso Galderisi, Domenico Vitale, Francesca Simonelli, Paolo Landolfo, Francesco Prisco, Mariorosario Masullo, Lucia Sacchetti

**Affiliations:** 1 CEINGE – Advanced Biotechnologies S. C. a R. L., Naples, Italy; 2 Department of Biochemistry and Medical Biotechnologies, University of Naples Federico II, Naples, Italy; 3 Department of Pediatrics, Second University of Naples, Naples, Italy; 4 SDN Foundation, Naples, Italy; 5 Department of Ophthalmology, Second University of Naples, Naples, Italy; 6 Department of Study of the Institutions and Territorial Systems, University of Naples “Parthenope”, Naples, Italy; University of Tampere, Finland

## Abstract

Maternally Inherited Diabetes and Deafness (MIDD) is a rare form of diabetes due to defects in mitochondrial DNA (mtDNA). 3243 A>G is the mutation most frequently associated with this condition, but other mtDNA variants have been linked with a diabetic phenotype suggestive of MIDD. From 1989 to 2009, we clinically diagnosed mitochondrial diabetes in 11 diabetic children. Diagnosis was based on the presence of one or more of the following criteria: 1) maculopathy; 2) hearing impairment; 3) maternal heritability of diabetes/impaired fasting glucose and/or hearing impairment and/or maculopathy in three consecutive generations (or in two generations if 2 or 3 members of a family were affected). We sequenced the mtDNA in the 11 probands, in their mothers and in 80 controls. We identified 33 diabetes-suspected mutations, 1/33 was 3243A>G. Most patients (91%) and their mothers had mutations in complex I and/or IV of the respiratory chain. We measured the activity of these two enzymes and found that they were less active in mutated patients and their mothers than in the healthy control pool. The prevalence of hearing loss (36% *vs* 75–98%) and macular dystrophy (54% *vs* 86%) was lower in our mitochondrial diabetic adolescents than reported in adults. Moreover, we found a hitherto unknown association between mitochondrial diabetes and celiac disease. In conclusion, mitochondrial diabetes should be considered a complex syndrome with several phenotypic variants. Moreover, deafness is not an essential component of the disease in children. The whole mtDNA should be screened because the 3243A>G variant is not as frequent in children as in adults. In fact, 91% of our patients were mutated in the complex I and/or IV genes. The enzymatic assay may be a useful tool with which to confirm the pathogenic significance of detected variants.

## Introduction

Maternally Inherited Diabetes and Deafness (MIDD) is a rare form of diabetes that accounts for up to 1% of all diabetes cases in Europeans and is due to defects in mitochondrial DNA (mtDNA) [1], [2]. In addition to maternal transmission of diabetes, the clinical features of MIDD are mainly neurosensorial deafness, followed by other mitochondrial disorders, myopathies, and macular dystrophy [1]. MIDD is often misdiagnosed as type 1, type 2 or monogenic diabetes [1], [3]. The absence of autoimmunity and obesity and the presence of maternal heritability, respectively, distinguish the latter three forms of diabetes from MIDD [1], [3]. Besides the frequently reported mtDNA 3243A>G mutation, whose functional significance has been evaluated [4], several other mtDNA variants have been associated with a diabetic phenotype suggestive of MIDD [5], [6]. However, few studies have explored the mitochondrial efficiency associated with detected mtDNA variants [7], [8]. Consequently, the pathogenic significance of many newly identified variants remains to be established.

The aim of this study was to look for DNA variants in the mitochondrial genome of a pediatric cohort with suspected mitochondrial diabetes from Southern Italy. Patients were selected for investigation based on stringent diagnostic criteria. The pathogenic role of the detected mutations was investigated using an informatics approach. We also spectrophotometrically evaluated the enzyme activity of the respiratory chain complexes I and IV mutated in the mtDNA of most of our patients and their mothers.

## Results

**Figure 1:**
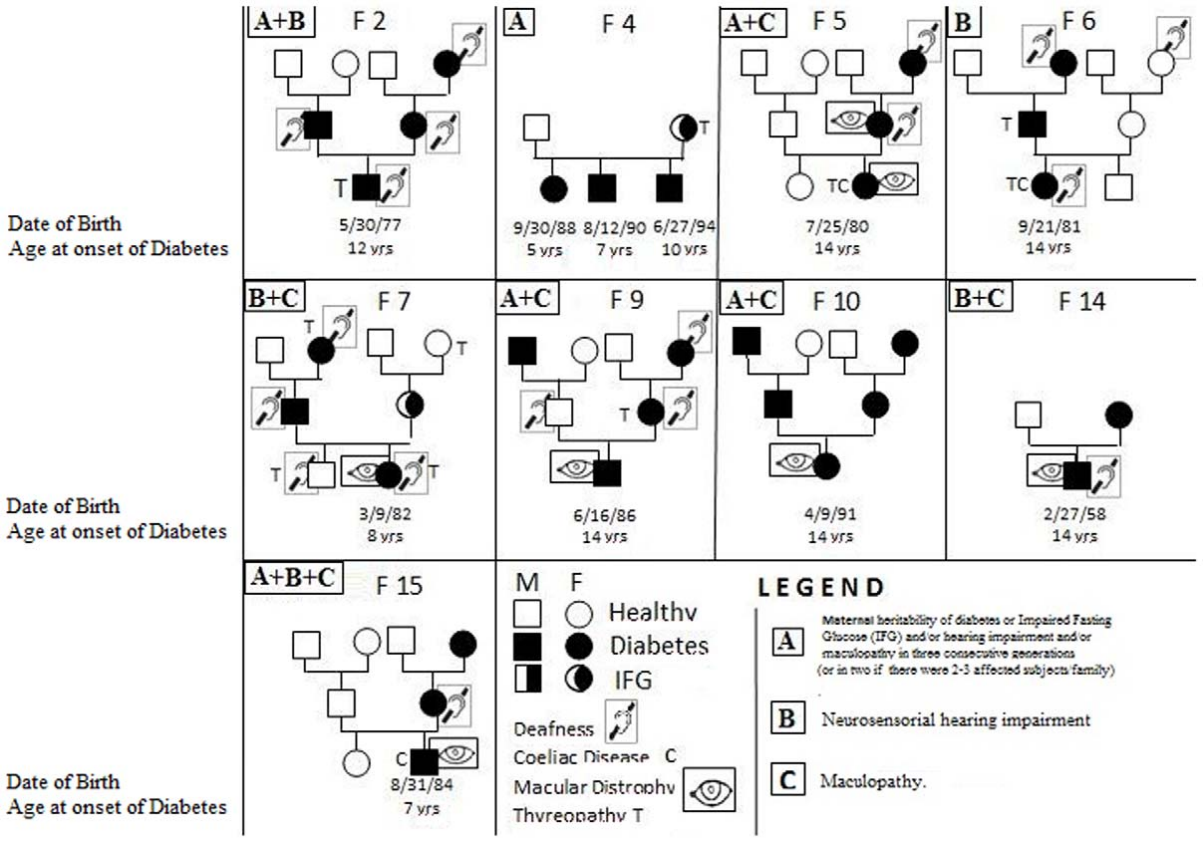
Familial (F) pedigrees of the suspected mitochondrial diabetes patients enrolled in the study. The inclusion criteria were: Diabetes+at least one of the following: A) maternal heritability of diabetes or Impaired Fasting Glucose (IFG) and/or hearing impairment and/or maculopathy in three consecutive generations (or in two if there were 2–3 affected subjects/family); B) neurosensorial hearing impairment; and C) maculopathy. In each square it's reported the presence of the criteria (A, B and/or C) in the probands.

The clinical and metabolic characteristics of the 11 patients with suspected mitochondrial diabetes are listed in Table 1 and their family pedigrees are shown in Figure 1. Median age at diabetes onset was 11 years (age range 5–14 years). Maternal inheritance of diabetes or IFG was documented in all but 1 patient: patient 6 who was affected by hypoacusia and had a maternal history of hypoacusia. All 11 patients needed insulin therapy and most were of normal weight (median *z* score 1.4). Macular dystrophy was the most frequent diabetes-associated disease (54%), but no patient had diabetic retinopathy, whereas neurosensorial hearing impairment was observed only in one-third of patients. Seven patients (64%) showed alterations of the muscle enzymes CK and/or LDH, 4 patients (36%) were affected by thyroiditis, and 9 patients had a maternal history of deafness and/or macular dystrophy and/or thyroiditis. Particularly, in addition to the clinical characteristics described in Fig. 1, we detected: high CK levels in patient 2; high CK, LDH and ALP levels in patients 4_1_, 4_2_, 4_3_; high LDH levels in patient 6; high ALP levels in patient 9; muscle pain in patient 14; and high CK levels and lactic acidosis in patient 15. Interestingly, HLA gene typing in the 11 patients revealed HLA-DQ2 and/or DQ8 molecules, and 3 were also affected by celiac disease (27%).

**Table 1:** Clinical and metabolic characteristics of pediatric patients from Southern Italy with suspected mitochondrial diabetes (n = 11)^a^.

Age at onset (years)	11.0 (5.0–14.0)
Ophthalmic diseases	
*-Macular dystrophy*	54%
*-Cataract*	18%
Hearing impairment	36%
Normal weight	82%
BMI (z score)^b^	1.4 (−0.9–2.4)
Insulin therapy	100%
Fasting Plasma glucose (mmol/L)	13.0 (8.0–21.2)
HbA_1c_ at diagnosis (%)	9.5 (6.3–14.0)
HbA_1c_ at diagnosis (mmol/mol)	80.33 (45.3–129.5)
Fasting C peptide at diagnosis (nmol/l)	0.06 (0.033–0.495)
CK (>174 U/L) and/or LDH (>190 U/L)	64%
Presence of HLA DQ2 and/or DQ8 alleles	100%
Thyroiditis	36%
Presence of celiac disease	27%
Maternal^c^ history of:	
*-Deafness*	45%
*-Maculopathy*	9%
*-Thyroiditis*	45%

a Continuous variables are reported as median (2.5^th^–97.5^th^ percentiles) and categorical variables as percentages;

b BMI z score = Body mass index z score;

c Mother and/or maternal relatives.

We sequenced the entire mitochondrial genome of the 11 patients, their mothers and 80 controls. The results were compared to the Revised Cambridge Reference Sequence (rCRS:NC_012920) [14]. We identified a total of 416 variants, among which 325 were detected only in controls, 58 were present in both controls and cases (Table S3), and 33 suspected mutations (4/33 novel) (Table 2) were present only in cases and their mothers. Among the suspected mutations detected only in patients, 22/33 were in the coding region (50% synonymous and 50% caused an amino acid change). Table 2 shows the main features (i.e.,the nucleotide variation, the relative amino acid substitution and its conservation across species, together with the bioinformatic-predicted role of the changed amino acid in the structure and/or function of the relative protein) of the variants detected in each patient. Each patient had from one to seven suspected mtDNA mutations. Table 2 also shows previously reported variants.

**Table 2:** Characteristics of suspected mtDNA mutations detected by sequence analysis in mitochondrial diabetic patients and their bioinformatic analysis.

Patient	Gene	Variant position	Amino acid change	ClustalW Conservation^a^	SIFT Score^b^	Poliphen prediction^c^	References^d^
2	NC7	8289–8290 insCCCCCTCTA					Novel Variant
	CO3	9803A>G	syn				Novel Variant
	CO3	9947G>A^e^	syn				31
4_1_, 4_2_, 4_3_	ND3	10373G>A	syn				-
	ND4	11447G>A	V230M	C	T/0.11	Benign	-
5	TL1	3243A>G^e^					32
6	HVII	293T>C					-
	ND5	12346C>T	H4Y	N	T/0.17	Benign	-
	CYB	15530 T>C	syn				-
7	HVII	385A>G					-
	TV	1664G>A					-
	ND2	5093T>C	syn				-
	ND2	5300C>T	syn				Novel Variant
	ATP8	8562C>G	P66R	N	T/0.44	Possibly Damaging	Novel Variant
	ND4	11928A>G	N390S	H	A/0.00	Benign	-
	ND1	4086C>T	syn				33
9	ND5	13135G>A	A267T	N	T/0.50	Benign	34
10	RNR1	960delC					35
	ND1	4024A>G	T240A	N	T/0.12	Benign	-
	ND6	14365C>T	V103M	C	A/0.01	Benign	-
	ND6	14582A>G	V31A	N	T/1	Benign	-
	HVI	16048G>A					-
14	CO3	9935T>C	syn				-
	CO3	9548G>A	syn				36
	ND4L	10685G>A	syn				-
	HVI	16137A>G					-
	HVI	16526G>A					-
15	RNR1	951G>A					-
	CO2	7762G>A	syn				-
	ND4	11253 T>C^e^	I165T	C	A/0.01	Possibly Damaging	37
	ND5	14002A>G	T556A	N	T/0.42	Benign	-
	ND6	14502T>C	I58V	C	T/0.36	Benign	-
	HVI	16354C>T					-

a Amino acid conservation evaluated with the ClustalW program, C: conserved/semi-conserved, N: not conserved, H: highly conserved.

b Score: T (tolerated: Score >0.05): The substitution is predicted to be functionally neutral, A (affected: score <0.05): The substitution is predicted to affect protein function.

c Evaluated with the Poliphen program (see Materials and Method for details). Benign: changes most likely lack a phenotypic effect; Possibly damaging: reflects a likelihood of affecting protein function or structure.

d When there is no reference, the variant was reported in MITOMAP, which is a human mitochondrial genome database http://www.mitomap.org.

e Heteroplasmic variants.

**Figure 2:**
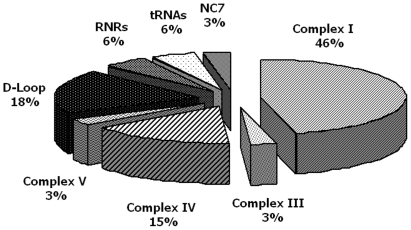
Percentage distribution in mitochondrial genome of suspected mutations detected in pediatric mitochondrial diabetic patients. Most diabetic associated variants (67%), detected by sequencing analysis, occurred in the coding region. The highest variant frequencies were observed in complex I (46%) and in complex IV (15%). In the non-coding region, the highest variant frequency was in the D-Loop (18%).

The 3243A>G variant in tRNA leucine, which is the mutation most frequently associated with MIDD, was present in only one of our patients (patient 5) at heteroplasmic level. The level of heteroplasmy was higher in the DNA of patient 5 than in his mother's DNA, in both swab and blood samples (Figure S1). qRT-PCR confirmed a higher level of heteroplasmy in the patient than in his mother (respectively 34% and 3%). The distribution (percentage) of suspected mutations in the non-coding and in the coding regions of mtDNA is reported in Figure 2. Most suspected mutations (67%) were in the coding region and those with the highest frequencies occurred in complex I (46%) (ND1: 4024A>G, 4086C>T; ND2:5093T>C, 5300C>T; ND3: 10373G>A; ND4: 11253T>C, 11447G>A, 11928A>G; ND4L:10685G>A; ND5:12346C>T, 13135G>A, 14002A>G; ND6:14365C>T, 14502T>C, 14582A>G), in complex-IV (15%) (CO2:7762G>A; CO3:9803A>G, 9935T>C, 9947G>A, 9548G>A) of the respiratory chain enzymes followed by complex III (3%) (CYB:15530T>C) and complex V (3%) (ATP8:8562C>G). Within the non-coding region, the highest suspected mutation frequency was in the D-Loop (18%) (HVI: 16048G>A, 16137A>G, 16354C>T, 16526G>A; HVII:293T>C, 385A>G), followed by RNRs (951G>A, 960delC) and tRNAs (TV:1664G>A; TL1:3243A>G) both 6%, and by the NC7 region (3%) (8289_8290insCCCCCTCTA).

Because almost all patients (10/11 = 91%) had suspected mutations in complex I and/or in complex IV, we measured the enzymatic activities of these two complexes to investigate if the variants identified were associated with impaired mitochondrial function in patients and in their mothers when samples were available (i.e., patients 2, 6, 9, 10 and 15). Patient 5 carried mutation 3243A>G and was not further investigated because the functional significance of this mutation has been well established (4). Table 3 shows the enzyme activities recorded in patients and their mothers after normalization first *vs* citrate synthase and then *vs* the healthy control pool. Residual complex I and/or complex IV enzyme activities were lower (below the detected biological variability of 40%) than in the control pool (set at 100%) in 4/5 patients and borderline in 1/5 patients. The enzyme activities in mothers were similar to those measured in their offspring except in mother 2 (a subject bearing 2 variants in complex IV, one of which at heteroplasmic level), in whom the residual enzyme activity was higher than in her son.

**Table 3:** Enzyme activities of the respiratory chain complexes I and IV evaluated in lymphocytes from mitochondrial diabetic patients (pt) and their mothers (m) bearing mtDNA variants in these complexes.

Sample ID	Pool^a^	pt2	m2	pt6	m6	pt9	m9	pt10	m10	pt15	m15
Mutated Complex		IV	IV	I	I	I	I	I	I	I,IV	I,IV
Complex I residual activity %^b^(nmol NADH oxidized min^−1^ mU^−1^ citrate synthase)	100	90	ND	39	12	64	65	32	33	52	20
Complex IV residual activity %^b^ (nmol Cytc oxidized min^−1^mU ^1^citrate synthase)	100	51	76	ND	ND	ND	ND	99	ND	46	56

a Pool is relative to 12 healthy control subjects.

b Residual activity (%) was obtained by normalization of the enzyme activity firstly *vs* citrate synthase and then *vs* the healthy cont rol pool. ND: Not determined.

## Discussion

Potentially pathogenetic mtDNA mutations have been identified in more than 5% of patients affected by type 2 diabetes [6], which suggests that the true prevalence of mitochondrial diabetes could be higher than usually reported in Europeans subjects 1% [1]. In our geographic area, the global incidence of diabetes, in the population under 15 years of age, is 6.4/100,000/year [19]. In our pediatric diabetology unit we diagnosed mitochondrial diabetes in 11/1600 children with a diabetic phenotype observed from 1989 to 2009, which corresponds to a prevalence of 0.6% of the diabetes. The study population included a “historical” case of 1972.

Most MDD studies [1], [20] started with the search for mutation 3243A>G in patients affected by both diabetes and deafness. Identification of the mutation prompted the investigation of the other common features (i.e., maculopathy and maternal heritability). Our approach was first to test all the diabetic patients of our Pediatric Diabetology Unit for maculopathy. Second, we carried out an audiometric examination of all patients positive for maculopathy or, if negative, in patients presenting maternal heritability of diabetes or IFG and/or hearing impairment and/or maculopathy in three consecutive generations (or in two generations if 2–3 members of the family were affected). This approach resulted in a lower incidence of deafness (36%) than previously reported, namely from 75% to 98% in 3243A>G-carriers with diabetes, with or without a maternal history of diabetes [1], [20], and 58% in type 2 diabetic patients bearing mtDNA variations [6]. The low incidence of deafness in our patients suggests that the designation of deafness as the main diagnostic criterion for mitochondrial diabetes may result in underestimation of the real prevalence of the disease. This is why, in our patients, we call the disorder “mitochondrial diabetes” rather than MIDD.

Macular dystrophy was present in 54% of our patients, which is also lower than the 86% reported in carriers of mutation 3243A>G [1], [20]. All our patients had DQ2 and/or DQ8 molecules that predispose to type 1 diabetes and to celiac disease. Intriguingly, celiac disease was detected in 27% (3/11 patients) of our suspected mitochondrial diabetic patients versus 1% of the general population [21] and versus 3%–6% of patients with type 1 diabetes [22]. As far as we are aware, this is the first report of an association between celiac disease and mitochondrial diabetes. Further investigations of mitochondrial function in celiac patients are required to verify the involvement of mtDNA variants in the pathogenesis or progression of the celiac disease. Notably, one-third of our mitochondrial diabetic patients had secondary thyroiditis, which has been previously reported in 3243A>G carriers in the presence of diabetes or other mitochondrial diseases [1], [23].

To our knowledge, there are no previous studies of mitochondrial diabetes in pediatric cohorts. In studies conducted in adults, young adults, in family case reports and in the MIDD 1 form, diabetes was usually diagnosed in patients aged between 16 and 43 years [1], [6], [24], [25]. The age at MIDD onset is also related to the heteroplasmy level of the mutations. In fact, in 3243A>G carriers with heteroplasmy levels of 34.5%, 14.9%, 14.6% and 5.9%, the age of MIDD onset was 15, 41, 44 and 65 years, respectively [26]. In agreement with these data, the age of diabetes onset was 14 years in our mitochondrial diabetic patient (patient 5) who had a heteroplasmy 3243A>G level of 34%. The heteroplasmic level of the mutation was higher in patient 5 than in his mother in both buccal cells and blood leucocytes, which is in agreement with previous reports [27]. This finding supports the concept that the heteroplasmy load in blood of 3243A>G declines with age [1], [28].

As mentioned above, the genetic analysis of MIDD usually focuses on the search for the 3243A>G mutation in selected diabetic patients affected by hearing loss; the entire mitochondrial genome is rarely screened [29], [30]. Sequence analysis of the whole mtDNA in our suspected mitochondrial diabetic pediatric patients and controls resulted in a high rate of mtDNA polymorphisms (a total of 383/416 variants, present also in controls). Consequently, it is important to ascertain the pathogenic significance of newly identified variants. Among the 33 suspected mutations, 11/22 (50%) of those occurring in the coding region caused an amino acid change. Using informatics we predicted a benign change by SIFT and/or Polyphen programs for 10/11 variants; however, contrasting results were generated for 4/11 variants. Only one of the four novel suspected mutations (8562C>G) detected in our population caused an amino acid change (P66R: a not conserved amino acid) in the ATP8 gene, the others being synonymous (2 variants) or present in the control region (1 variant).

Most of the suspected mutations detected in our cohort have been described previously, often in a single patient or family, in association with diabetes, with other mitochondriopathies (mitochondrial encephalomyopathy, lactic acidosis and stroke-like episodes: MELAS, Leber's hereditary optic neuropathy: LHON), with hearing loss [31]–[37], with cancer and with Parkinson disease or in population studies (MITOMAP: A Human Mitochondrial Genome Database:http://www.mitomap.org). However, the true clinical significance of these suspected mutations, apart from 3243A>G [4], has been scarcely investigated [7], [8]. Given the highly polymorphic patterns detected in our patients, each usually bearing more than one variant (range: 1–7), and each variant being present once in the cohort, we measured the enzyme activities of complexes I and IV, where most of the variants occurred. The residual enzyme activity of the relative complex was lower in mitochondrial diabetic patients (5 patients) and in their mothers (complex I 12–65% and complex IV 46–76%) than in the healthy control pool set at 100%, although there was no correlation between the diabetic phenotype and the level of the residual enzyme activity in our patients. Intriguingly, patient 6 was affected by diabetes and deafness, her grandmother was affected by deafness and her father by diabetes. Although we detected some potentially pathogenic mtDNA variants and a reduced enzyme activity of complex I in both patient 6 and her mother, we cannot exclude that other genetic factors could have contributed to the diabetic phenotype of this patient. In fact, the phenotype of a pathogenic mtDNA mutation, or the severity of an mtDNA mutation that may not be pathological in some cases, could be influenced by the mitochondrial DNA haplogroup [38]. In addition, the genetic instability of mtDNA heteroplasmic mutations in the patient's somatic tissues [39], or the nuclear background, by nuclear modifiers, may also play a role in determining mtDNA mutation pathogenicity [40].

In conclusion, mitochondrial diabetes should be considered a complex syndrome with several phenotypic variants. Deafness is not an essential component of the disease in children. Investigations of patients should include the study of the entire mtDNA because the 3243A>G variant is not as frequent in children as in adults. In fact, 91% of our patients were mutated in the complex I and/or IV genes. The enzymatic assay may be a useful tool with which to measure the mitochondrion dysfunction associated with detected mtDNA variants.

## Materials and Methods

### Patients and controls

Sixteen patients (including 3 brothers), with suspected mitochondrial diabetes were enrolled from among the diabetic population attending the Department of Pediatrics of the Second University of Naples (Italy) (15/16 from 1989 to 2009 and 1/16, a historical case in 1972). Controls (10 affected by type 1 diabetes and 70 healthy controls) were recruited at DASMELAB/CEINGE–Advanced Biotechnology/University of Naples Federico II. All patients (54% males), their mothers, and controls had lived in Southern Italy for at least 2–3 generations. All the diabetic children were screened for maculopathy by ophtalmoscopic examination. Inclusion criteria for suspected mitochondrial diabetes, in addition to the presence of diabetes defined according to the American Diabetes Association (ADA) [9], were: 1) maternal heritability of diabetes or impaired fasting glucose (IFG) and/or hearing impairment and/or maculopathy in three consecutive generations (or in two generations if 2 or 3 members of the family were affected); 2) neurosensorial hearing impairment; and 3) maculopathy. At least one or more of these criteria were required for enrollment in the study (Figure 1). Audiometric examination was performed in all patients with maculopathy, and in patients without maculopathy if they fulfilled one of the above-indicated clinical criteria. A cut-off point of 250 Hz with a slope of 24 dB/oct was considered diagnostic of hearing impairment [10]. The ophthalmoscopic examinations were performed according to standardized procedures [11], [12]. Age at disease onset, need of insulin therapy, levels of fasting C peptide, and type 1 diabetes autoantibodies were also recorded. All pedigrees were verified from the patients' records by expert pediatricians and in cooperation with the family doctor. Patients with suspected mitochondrial diabetes were also typed for Human Leukocyte Antigen (HLA) -DRB1(*03/*04/*07/*11), DQA1 (*02/*03/*05), and DQB1(*02–*06) alleles (Histotype Special Medium Resolution and Histotype DQB Low SSP Kits- BAG Healthcare) to identify HLA alleles predisposing to type 1 diabetes and/or to other autoimmune diseases. To determine whether our patients were affected by the autoimmune diseases most frequently associated with type 1 diabetes (thyroiditis and celiac disease), we carried out the following immunofluorometric or immunoenzymatic assays: free triiodothyronine (FT3), free thyroxine (FT4), thyroid-stimulating hormone (TSH), thyroglobulin (TG), anti-thyroglobulin (Anti-TG), anti-peroxidase (Anti-TPO) antibodies and IgA-IgG anti-gliadin antibodies (AGA) and IgA transglutaminase (TGase) antibodies. The presence of celiac disease in serology-positive participants was confirmed by total or subtotal villous atrophy at biopsy examination. We also screened our patients for myopathy (muscle enzymes alterations) by measuring creatine kinase (CK)>174 U/L and/or lactate dehydrogenase (LDH)>190 U/L) levels. A detailed family history, and anthropometric and clinical data were collected on a standard case-record form.

The presence of hypertension, defined as blood pressure in excess of the 90° percentile in children [13] was also explored. A fasted blood sample was collected from all patients at the first clinical examination for routine biochemical investigations: glucose, C-peptide, glycated hemoglobin (HbA_1C_), islet cell antibody (ICA), Anti-glutamate decarboxylase (GAD), protein tyrosine phosphatase (IA2), insulin auto antibody (IAA), CK, LDH, creatinine, which were determined with routine procedures. Based on biochemical findings, five of 16 suspected mitochondrial diabetic patients were diagnosed as type 1 diabetes (high positivity for all the tested diabetes type 1 autoimmune markers), and were excluded from the study. Although autoimmune markers do not rule out a mitochondrial form of diabetes, in this preliminary study we preferred to avoid any factor that could interfere with the pathogenetic mechanism of a supposed “mitochondrial form”. A peripheral blood+EDTA sample was also collected from patients, their mothers and controls to obtain DNA samples for mtDNA sequence analysis. The mtDNA analysis was also performed in patients with suspected mitochondrial diabetes and their mothers on buccal cells collected by swab. Written informed consent was obtained from all recruited subjects, in the case of children, consent was obtained from their parents, and the study was approved by the Ethics Committee of the Faculty of Medicine of the Second University of Naples (Italy). The study was performed according to the Helsinki declaration.

### DNA extraction and mtDNA sequencing

Genomic DNA was extracted with the Kit-Nucleon-BACC2 (Illustra DNA-Extraction Kit-BACC2-GE Healthcare, UK) and stored at +4°C. The primers used to amplify by PCR the mtDNA were chosen by the PRIMER 3 program (http://frodo.wi.mit.edu/primer3/) and were selected to generate two overlapping fragments encompassing the whole mitochondrial genome. Primers are listed in Table S1. The mtDNA was amplified by long PCR using GeneAmp PCR System 9700 (Applied-Biosystems, Foster City, CA, USA). The long PCR mixture and conditions are detailed in Methods S1. The PCR fragments were examined by electrophoresis to assess yield and purity, then amplicons were purified over affinity spin columns (Qiaquick-PCR purification Kit, Qiagen Hilden, Germany). The whole mitochondrial genome was then sequenced with the BigDye Terminator v3.1 cycle sequencing method on the ABI-Prism 3730 Genetic Analyzer (Applied-Biosystems) by using 32 forward primers, summarised in Table S2, and analyzed using the SeqScape program (v2.5 Applied-Biosystems) to compare the mtDNA sequences of patients and controls with the revised Cambridge Reference Sequence (rCRS) [14].

### Real-time quantitative PCR

We used the TaqMan system (7900HT Fast-Real-Time-system; Applied-Biosystems) to evaluate by real-time quantitative PCR (qRT-PCR) the level of heteroplasmic 3243A>G mutation detected in one patient and his mother. Primers and Real-time quantitative PCR conditions were detailed in Methods S2. To quantify total and mutant mtDNA, standard curves were constructed using plasmids with the wild-type (WT) and the mutant mtDNA fragments respectively. The ratio between total mtDNA and mutant mtDNA was calculated in each sample.

### Bioinformatic analysis

We used the Sorting Intolerant from Tolerant (SIFT) (http://sift.jcvi.org/), Polymorphism Phenotyping (PolyPhen) (http://genetics.bwh.harvard.edu/pph) and ClustalW http://www.ebi.ac.uk/Tools/clustalw2/index.html) programs to predict the pathogenicity of the detected missense suspected mutations. The evaluation included amino acid conservation across species and the role of the changed amino acid in the structure and/or in the function of the relative protein.

### Evaluation of the enzyme activities of complexes I and IV of the respiratory chain

Lymphocytes from mitochondrial diabetic patients, their mothers, and 12 healthy controls were first isolated from fresh peripheral blood+EDTA (10 ml) and then separated on Ficoll medium using Ficoll Paque plus reagent (GE Healthcare, Waukesha, WI, USA) as previously described [15]. Briefly, the blood was diluted by the addition of an equal volume of PBS (Sigma-Aldrich Corp., St. Louis, MO, USA), then aliquots of 7 mL of this mixture were layered over 3 mL of Ficoll Paque and centrifuged at 400 g, at 18°C for 30 min. The mononuclear cell fraction was removed, diluted in PBS (1∶10) and centrifuged. The pellet was washed with 5 mL of PBS and lymphocytes were counted with the automated Analyzer Coulter LH 750 (Beckman Coulter Inc., Fullerton, CA, USA) and finally aliquots of 2.5×10^6^ lymphocytes were resuspended in an ice-cold buffer (SHE-PIM) containing 250 mmol/l sucrose, 10 mmol/l HEPES pH 7.4 and 1 mmol/l EDTA (SHE) supplemented with a protease inhibitor mixture (PIM) (Complete, EDTA-free, Roche Diagnostics, Mainheim, Germany). These aliquots of lymphocytes were rapidly frozen in liquid nitrogen and stored at −80°C.

#### Sample preparation for the enzyme assays

Cells were permeabilized by four freeze/thawing cycles. The protein content of the aliquots of lymphocytes was determined by the Bio-Rad Protein Assay (Biorad Laboratories, GmbH, Munchen, Germany) using BSA as standard.

NADH: Ubiquinone-oxidoreductase (Complex-I) Activity: Complex-I activity was measured as previously described [16] by monitoring the oxidation of NADH to NAD^+^ at 340 nm at 37°C, using a Cary 1E Spectrophotometer (Varian), equipped with an electronic temperature controller, and a molar extinction coefficient of 6220 M^−1^cm^−1^. The baseline absorbance variation was determined by adding an appropriate amount of permeabilized lymphocytes (usually 2.5×10^6^–5.0×10^6^). Therefore, the reaction started with the addition of 50 µmol/l Coenzyme-Q (CoQ1) in the absence or in the presence of 5 µmol/l rotenone, a specific complex I inhibitor, and was monitored for an additional 3–5 min. Under these conditions, the rate of NADH oxidation measured in the absence of rotenone corresponded to the total NADH-CoQ oxidoreductase activity, whereas that measured in its presence reflected the rotenone-insensitive NADH-CoQ oxidoreductase activity (RINQ), which is not associated to complex I activity. Therefore, the activity of complex I can be derived subtracting the rate of RINQ from the total activity and is expressed as nmol of NADH oxidized min^−1^ mg^−1^ of protein.

#### Cytochrome c oxidase (complex IV) activity

Complex IV activity was determined by monitoring the oxidation of reduced cytochrome-*c* (rCyt-*c*) at 550 nm (molar extinction coefficient 29500 M^−1^ cm^−1^). rCyt-*c* was prepared from commercially available oxidised Cytochrome c (Sigma Aldrich) by reduction with dithiothreitol (DTT) and quantification as indicated by the manufacturer. The assay mixture, prepared in 10 mmol/l potassium phosphate, pH 7.4, contained 1.5% n-dodecyl-β-D-maltoside, which is required to maximize complex IV activity [17], and 25 µmol/l rCyt-*c*. After reading the baseline activity, the reaction was started by adding appropriate amounts of permeabilized lymphocytes (usually 2.0×10^4^–3.0×10^4^ cells), and followed by measuring the decrease of the absorbance at 550 nm for an additional 2–3 min. Complex IV activity was expressed as nmol Cyt-*c* oxidized min^−1^ mg^−1^ of protein. Both complex I and complex IV activities were normalized to citrate synthase activity, which is an index of mitochondrial mass, as previously reported [17], to correct for any differential mitochondrial content due to mitochondrial dysfunction [18]. Finally, the residual percent activities of complex I and complex IV in both mitochondrial diabetic patients and their mothers were obtained by comparing their activities with that measured in a healthy control pool set at 100%. The biological variability of enzyme activities in the samples used for the control pool was 40%, in agreement with the previously reported value [16]. To check the quality of our assay, we also verified the enzyme activity of two un-mutated complexes in two patients (patients 2 and 10) and in the father of a diabetic patient (data not shown). The residual enzyme activities in these controls ranged from 87% to 99%.

### Statistical analysis

The statistical analysis of biochemical and general data from patients was carried out using PASW 18.0 software version (SPSS Inc., Chicago, IL, USA). The Kolmogorov-Smirnov test was used to evaluate the distribution of continuous variables (age at onset, BMI Z score, fasting plasma glucose, glycated haemoglobin, fasting C peptide at diagnosis) that were expressed as median (2.5^th^–97.5^th^ percentiles). The categorical variables were reported as percentage.

## Supporting Information

Figure S1 **Detection of 3243A>G mitochondrial mutation by sequence analysis.** Sequence analysis of swab and blood mtDNA from patient 5 (pt5) and from her mother (m5) showing the heteroplasmic 3243A>G mutation. Levels of heteroplasmy were higher in mtDNA from pt5 than in mtDNA from m5 in both swab and blood samples.
(PDF)

Methods S1 **Mixture and conditions for Long PCR of mtDNA.**
(PDF)

Methods S2 **Primers and Real-time quantitative PCR conditions.**
(PDF)

Table S1 **Primers sequence for Long PCR of mtDNA.** Table shows the two primers pair used for the amplification of the entire mtDNA.
(PDF)

Table S2 **Primers sequence for mtDNA sequencing.** Table shows the primers used for the sequencing of the mtDNA amplification products.
(PDF)

Table S3 **MtDNA Variants detected in control subjects or both in control subjects and patients.** Table reports the 325 mtDNA variants detected only in our controls subjects and the 58 variants detected both in control subjects and patients. For each variant is reported the mitochondrial region, the nucleotide and amino acid change and the relative frequency.
(PDF)

## References

[1] Murphy R, Turnbull DM, Walker M, Hattersley AT (2008). Clinical features, diagnosis and management of maternally inherited diabetes and deafness (MIDD) associated with the 3243A>G mitochondrial point mutation.. Diabet Med.

[2] Maassen JA, Jahangir Tafrechi RS, Janssen GM, Raap AK, Lemkes HH (2006). New insights in the molecular pathogenesis of the maternally inherited diabetes and deafness syndrome.. Endocrinol Metab Clin North Am.

[3] Murphy R, Ellard S, Hattersley AT (2008). Clinical implications of a molecular genetic classification of monogenic β–cell diabetes.. Nat Clin Pract Endocrinol Metab.

[4] Maassen JA, 'T Hart LM, Van Essen E, Heine RJ, Nijpels G (2004). Mitochondrial diabetes: molecular mechanisms and clinical presentation.. Diabetes.

[5] Maechler P, Wollheim CB (2001). Mitochondrial function in normal and diabetic beta–cells.. Nature.

[6] Crispim D, Estivalet AA, Roisenberg I, Gross JL, Canani LH (2008). Prevalence of 15 mitochondrial DNA mutations among type 2 diabetic patients with or without clinical characteristics of maternally inherited diabetes and deafness.. Arq Bras Endocrinol Metab.

[7] Mariotti C, Tiranti V, Carrara F, Dallapiccola B, Di Donato S (1994). Defective respiratory capacity and mitochondrial protein synthesis in transformant cybrids harboring the tRNA(Leu(UUR)) mutation associated with maternally inherited myopathy and cardiomyopathy.. J Clin Invest.

[8] Malfatti E, Bugiani M, Invernizzi F, de Souza CF, Farina L (2007). Novel mutations of ND genes in complex I deficiency associated with mitochondrial encephalopathy.. Brain.

[9] American Diabetes Association (2010). Diagnosis and Classification of Diabetes Mellitus.. Diabetes Care.

[10] Sawada S, Takeda T, Kakigi A, Saito H, Suehiro T (1997). Audiological findings of sensorineural deafness associated with a mutation in the mitochondrial DNA.. Am J Otol.

[11] Massin P, Virally-Monod M, Vialettes B, Paques M, Gin H (1999). Prevalence of macular pattern dystrophy in Maternally Inherited Diabetes and Deafness. GEDIAM Group.. Ophthalmology.

[12] Massin P, Guillausseau PJ, Vialettes B, Paquis V, Orsini F (1995). Macular pattern dystrophy associated with a mutation of mitochondrial DNA.. Am J Ophthalmol.

[13] Chobanian AV, Bakris GL, Black HR, Cushman WC, Green LA (2003). Seventh report of the Joint National Committee on Prevention, Detection, Evaluation, and Treatment of High Blood Pressure.. Hypertension.

[14] Andrews RM, Kubacka I, Chinnery PF, Lightowlers RN, Turnbull DM (1999). Reanalysis and revision of the Cambridge reference sequence for human mitochondrial DNA.. Nat Genet.

[15] Fuss IJ, Kanof ME, Smith PD, Zola H (2009). Isolation of whole mononuclear cells from peripheral blood and cord blood.. Curr Protoc Immunol.

[16] de Wit LE, Spruijt L, Schoonderwoerd GC, de Coo IF, Smeets HJ (2007). A simplified and reliable assay for complex I in human blood lymphocytes.. J Immunol Methods.

[17] Kirby DM, Thorburn DR, Turnbull DM, Taylor RW, Pon LA, Schon EA (2007). Biochemical assays of respiratory chain complex activity.. Methods in cell biology, 2nd ed.

[18] Boushel R, Gnaiger E, Schjerling P, Skovbro M, Kraunsøe R (2007). Patients with type 2 diabetes have normal mitochondrial function in skeletal muscle.. Diabetologia.

[19] Prisco F, Vicedomini D, Iafusco D, De Felice E, Amodeo BM (1996). Incidence of IDDM in the Campania Region, Italy.. Diabetes Care.

[20] Guillausseau PJ, Massin P, Dubois-LaForgue D, Timsit J, Virally M (2001). Maternally inherited diabetes and deafness: a multicenter study.. Ann Intern Med.

[21] Kagnoff MF (2007). Celiac disease: pathogenesis of a model immunogenetic disease.. J Clin Invest.

[22] Dubé C, Rostom A, Sy R, Cranney A, Saloojee N (2005). The prevalence of celiac disease in average-risk and at-risk Western European populations: a systematic review.. Gastroenterology.

[23] Balestri P, Grosso S (2000). Endocrine disorders in two sisters affected by MELAS syndrome.. J Child Neurol.

[24] Hosszúfalusi N, Karcagi V, Horváth R, Palik E, Várkonyi J (2009). A detailed investigation of maternally inherited diabetes and deafness (MIDD) including clinical characteristics, C- peptide secretion, HLA-DR and –DQ status and autoantibody pattern.. Diabetes Metab Res Rev.

[25] Guillausseau PJ, Dubois-Laforgue D, Massin P, Laloi-Michelin M, Bellanné-Chantelot C (2004). GEDIAM, Mitochondrial Diabetes French Study Group. Heterogeneity of diabetes phenotype in patients with 3243 bp mutation of mitochondrial DNA (Maternally Inherited Diabetes and Deafness or MIDD).. Diabetes Metab.

[26] Zhang S, Tong AL, Zhang Y, Nie M, Li YX (2009). Heteroplasmy level of the mitochondrial tRNaLeu (UUR) A3243G mutation in a Chinese family is positively associated with earlier age-of onset and increasing severity of diabetes.. Chin Med Sci J.

[27] Maassen JA, Janssen GM, 't Hart LM (2005). Molecular mechanisms of mitochondrial diabetes (MIDD).. Ann Med.

[28] Laloi-Michelin M, Meas T, Ambonville C, Bellanné-Chantelot C, Beaufils S (2009). Mitochondrial Diabetes French Study Group. The clinical variability of maternally inherited diabetes and deafness is associated with the degree of heteroplasmy in blood leukocytes.. J Clin Endocrinol Metab.

[29] Choo-Kang AT, Lynn S, Taylor GA, Daly ME, Sihota SS (2002). Defining the importance of mitochondrial gene defects in maternally inherited diabetes by sequencing the entire mitochondrial genome.. Diabetes.

[30] Alcolado JC, Laji K, Gill-Randall R (2002). Maternal transmission of diabetes.. Diabet Med.

[31] Hanna MG, Nelson IP, Morgan-Hughes JA, Wood NW (1998). MELAS: a new disease associated mitochondrial DNA mutation and evidence for further genetic heterogeneity.. J Neurol Neurosurg Psychiatry.

[32] Howes T, Madden C, Dasgupta S, Saeed S, Das V (2008). Role of mitochondrial variation in maternally inherited diabetes and deafness syndrome.. J Laryngol Otol.

[33] Chen J, Yang L, Yang A, Zhu Y, Zhao J (2007). Maternally inherited aminoglycoside-induced and nonsyndromic hearing loss is associated with the 12S rRNA C1494T mutation in three Han Chinese pedigrees.. Gene.

[34] Qian Y, Zhou X, Hu Y, Tong Y, Li R (2005). Clinical evaluation and mitochondrial DNA sequence analysis in three Chinese families with Leber's hereditary optic neuropathy.. Biochem Biophys Res Commun.

[35] Elstner M, Schmidt C, Zingler VC, Prokisch H, Bettecken T (2008). Mitochondrial 12S rRNA susceptibility mutations in aminoglycoside-associated and idiopathic bilateral vestibulopathy.. Biochem Biophys Res Commun.

[36] Horváth R, Reilmann R, Holinski-Feder E, Ringelstein EB, Klopstock T (2008). The role of complex I genes in MELAS: a novel heteroplasmic mutation 3380G>A in ND1 of mtDNA.. Neuromuscul Disord.

[37] Leo-Kottler B, Luberichs J, Besch D, Christ-Adler M, Fauser S (2002). Leber's hereditary optic neuropathy: clinical and molecular genetic results in a patient with a point mutation at np T11253C (isoleucine to threonine) in the ND4 gene and spontaneous recovery.. Graefes Arch Clin Exp Ophthalmol.

[38] Gutiérrez Cortés N, Pertuiset C, Dumon E, Börlin M, Herbert-Chatelain E (2012). Novel mitochondrial DNA mutations responsible for maternally inherited non-syndromic hearing loss.. Hum Mutat.

[39] Bannwarth S, Abbassi M, Valéro M, Fragaki K, Dubois N (2011). A novel instable mutation in mitochondrial DNA responsible for maternally inherited diabetes and deafness.. Diabetes Care.

[40] Carelli V, Giordano C, d'Amati G (2003). Pathogenic expression of homoplasmic mtDNA mutations needs a complex nuclear-mitochondrial interaction.. Trends Genet.

