# Supplementary material for: Mitochondrial Diabetes in Children: Seek and You Will Find It
Source: PLoS One. 2012 Apr 19;7(4):e34956. doi: 10.1371/journal.pone.0034956 (PMC3334935; doi:10.1371/journal.pone.0034956)
Supplement: Figure S1 — Detection of 3243A>G mitochondrial mutation by sequence analysis. Sequence analysis of swab and blood mtDNA from patient 5 (pt5) and from her mother (m5) showing the heteroplasmic 3243A>G mutation. Levels of heteroplasmy were higher in mtDNA from pt5 than in mtDNA from m5 in both swab and blood samples. (PDF) [file pone.0034956.s001.pdf]

Figure S1. Sequence analysis showing the heteroplasmic 3243A>G mtDNA mutation detected in the patient 5 (pt 5) and her mother (m 5) (swab and blood).

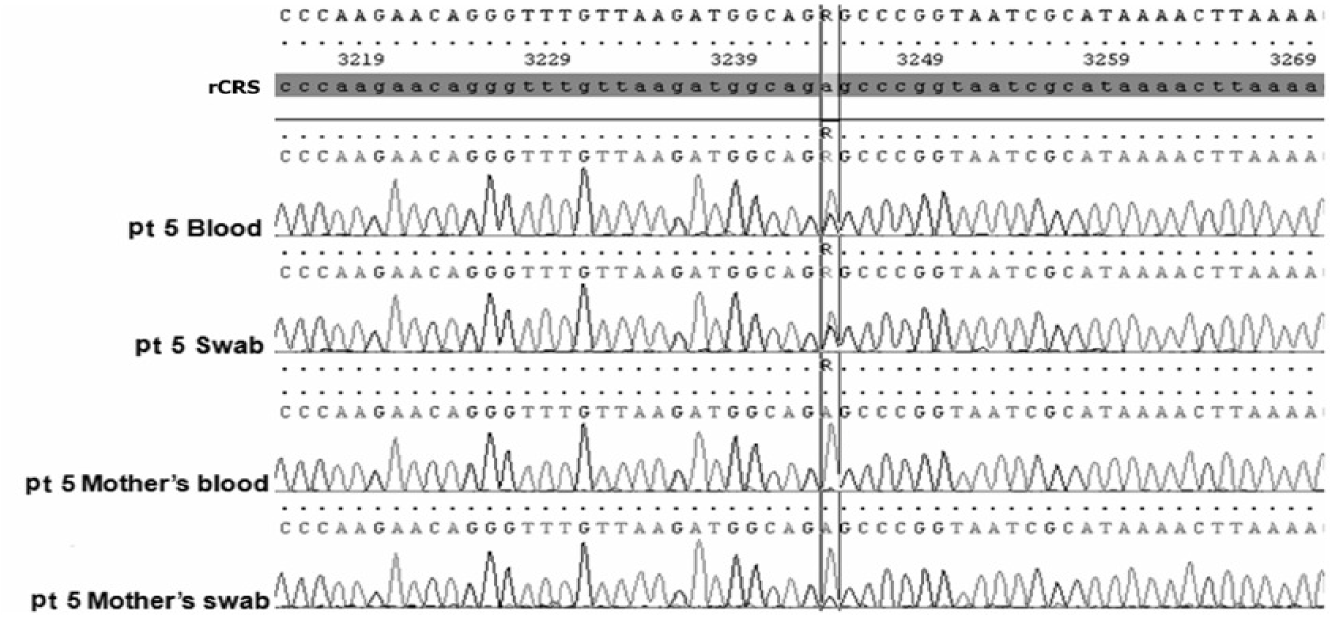

Levels of heteroplasmy of 3243A>G mutation, displayed for each sample between the two lines, were higher in mtDNA from pt 5 than in mtDNA from m5 in both swab and blood samples. rCRS: revised Cambridge Reference Sequence; R: unidentified nucleotide between adenosine and guanosine.
