# Supplementary material for: Mitochondrial Diabetes in Children: Seek and You Will Find It
Source: PLoS One. 2012 Apr 19;7(4):e34956. doi: 10.1371/journal.pone.0034956 (PMC3334935; doi:10.1371/journal.pone.0034956)
Supplement: Methods S1 — Mixture and conditions for Long PCR of mtDNA. (PDF) [file pone.0034956.s002.pdf]

Methods S1. Mixture and conditions for Long PCR of mtDNA

The entire mtDNA was amplified in two fragments, using two primer pairs. In a final volume of 50 µL, the PCR mixture contained: 0.4 µmol/l of each primer, 1.5x of cloned PFU reaction buffer, 500 µmol/l of each deoxynucleotide triphosphate, and 2.5U of *Taq*-DNA polymerase (Applied-Biosystems), 1.25U of PFU Turbo DNA polymerase (Stratagene) and 50 ng of genomic DNA. The PCR conditions were: first, one cycle at 92 °C for 1 min, followed by 35 cycles at 56°C for 20 s, at 68°C for 9 min, and at 90°C for 10 s, and finally, one cycle at 68°C for 15 min followed by cooling to 4°C.
