# Supplementary material for: Mitochondrial Diabetes in Children: Seek and You Will Find It
Source: PLoS One. 2012 Apr 19;7(4):e34956. doi: 10.1371/journal.pone.0034956 (PMC3334935; doi:10.1371/journal.pone.0034956)
Supplement: Methods S2 — Primers and Real-time quantitative PCR conditions. (PDF) [file pone.0034956.s003.pdf]

Methods S2. Primers and Real-time quantitative PCR conditions

We used two set of primers, one specific for the 3243A>G mutation (5'-ATTAAAGTCCTACGTGATC-3' at 3048nt–3066nt and 5'-ATGCGATTACCGGGCC-3' at 3258nt–3243nt) and the other to amplify the entire mtDNA (5'-GCCTTCCCCCGTAAATGATAT-3' at 3163nt–3183nt and 5' GAAGAGGAATTGAACCTCTGACTG-3' at 3298nt–3275nt). The nucleotide sequence of the TaqMan probe was TGCCATCTTAACAAACCCTGTTCTTGGGTT (3241nt–3213nt). PCR was performed for 40 cycles at 95°C for 15 s, at 51°C for 10 s and at 57°C for 1 min.
