# Supplementary material for: Mitochondrial Diabetes in Children: Seek and You Will Find It
Source: PLoS One. 2012 Apr 19;7(4):e34956. doi: 10.1371/journal.pone.0034956 (PMC3334935; doi:10.1371/journal.pone.0034956)
Supplement: Table S1 — Primers sequence for Long PCR of mtDNA. Table shows the two primers pair used for the amplification of the entire mtDNA. (PDF) [file pone.0034956.s004.pdf]

Table S1 Primers sequence for Long PCR of mtDNA

Name	Primer sequence 5'-3'/PCR	Nucleotide Position
mt1 forward	CACAGCCACTTTCCACACAG	257
mt1 reverse	TTTGCTCCACAGATTTCAGA	8175
mt2 forward	GGCGGACTAATCTTCAACTC	7926
mt2 reverse	TTGTTTATGGGGTGATGTGA	630
