# Supplementary material for: Mitochondrial Diabetes in Children: Seek and You Will Find It
Source: PLoS One. 2012 Apr 19;7(4):e34956. doi: 10.1371/journal.pone.0034956 (PMC3334935; doi:10.1371/journal.pone.0034956)
Supplement: Table S2 — Primers sequence for mtDNA sequencing. Table shows the primers used for the sequencing of the mtDNA amplification products. (PDF) [file pone.0034956.s005.pdf]

Table S2 Primers sequence for mtDNA sequencing

Name	Primer sequence 5'-3'/Sequencing	Nucleotide Position
1	GCTTCTGGCCACAGCACT	316
2	GCATCAAGCACGCAGCAA	755
3	ACCGCCATCTTCAGCAAACC	1260
4	GCTAAGACCCCCGAAACCAG	1894
5	CGATGGTGCAGCCGCTATTA	3009
6	TTACCGGGCTCTGCCATCTT	3252
7	GCGAGCAGTAGCCCAAACAA	3705
8	CCCATTACAATCTCCAGCAT	4228
9	CCGGACAATGAACCATAACCAA	4711
10	AGAAGCCCCGGCAGGTTT	5759
11	CGCTAACCGGCTTTTTGC	5236
12	ATATGGCGTTTCCCCGCATA	6175
13	TGGCTTCCTAGGGTTTATCGTG	6743
14	CTGAGAACCAAAATGAACGA	8515
15	GGCGGACTAATCTTCAACTC	7926
16	TTTGCTCCACAGATTTCAGA	8175
17	GCGCCACCCTAGCAATATCA	9053
18	CTGAGCTCACCATAGTCT	9641
19	ATCCACCCCTTACGAGTG	10157
20	CCCCACCTTGGCTATCATCA	11140
21	CCAAATGCCCCTCATTTACA	10466
22	CCTATCCCTCAACCCCGACA	12097
23	CCCCATCGCTGGGTCAATAG	11428
24	CAGCAGTCTGCGCCCTTACA	13199
25	CCCAAACAACCCAGCTCTCC	12548
26	CTCCCGAATCAACCCTGACC	14263
27	ACCTCCCTGACAAGCGCCTA	13582
28	TGCACGAAACGGGATCAAAC	15345
29	TTCTCGCACGGACTACAACCA	14676
30	GCCGCAGACCTCCTCATTCT	15731
31	GGTCCCTTGACCACCATCCT	16390
32	GCTTCTGGCCACAGCACT	316
