# Supplementary material for: Mitochondrial Diabetes in Children: Seek and You Will Find It
Source: PLoS One. 2012 Apr 19;7(4):e34956. doi: 10.1371/journal.pone.0034956 (PMC3334935; doi:10.1371/journal.pone.0034956)
Supplement: Table S3 — MtDNA Variants detected in control subjects or both in control subjects and patients. Table reports the 325 mtDNA variants detected only in our controls subjects and the 58 variants detected both in control subjects and patients. For each variant is reported the mitochondrial region, the nucleotide and amino acid change and the relative frequency. (PDF) [file pone.0034956.s006.pdf]

Table S3 MtDNA variants and their percentage detected in control subjects or both in control subjects and Mitochondrial Diabetic patients

mtDNA				
**Region**	**Nucleotide change**	**Amino acid change**	**Mitochondrial Diabetic patients n (%)**	**Control subjects n (%)**
D-Loop	72T>C		2 (18.2)	2 (2.5)
D-Loop	73A>G		2 (18.2)	47 (58.8)
D-Loop	93A>G			2 (2.5)
D-Loop	114C>T			2 (2.5)
D-Loop	143G>A			3 (3.7)
D-Loop	146T>C		1 (9.1)	8 (10.0)
D-Loop	150C>T			11 (13.7)
D-Loop	151C>T			3 (3.7)
D-Loop	152T>C		3 (27.3) [a]	15 (18.8)
D-Loop	153A>G			6 (7.5)
D-Loop	183A>G			1 (1.2)
D-Loop	185G>A			3 (3.7)
D-Loop	189A>G			3 (3.7)
D-Loop	194C>T			2 (2.5)
D-Loop	195T>C		1 (9.1)	14 (17.5)
D-Loop	198C>T			2 (2.5)
D-Loop	199T>C			2 (2.5)
D-Loop	200A>G			1 (1.2)
D-Loop	204T>C			5 (6.2)
D-Loop	207G>A		1 (9.1)	3 (3.7)
D-Loop	210A>G			1 (1.2)
D-Loop	225G>A			4 (5.0)
D-Loop	228G>A			4 (5.0)
D-Loop	250T>C			2 (2.5)
D-Loop	263A>G		11 (100)	71 (88.7)
**D-Loop**	**281A>C**			**1 (1.2)**
D-Loop	294T>C			1 (1.2)
D-Loop	295C>T			7 (8.7)
D-Loop	310T>C		3 (27.3)	36 (45.0)
D-Loop	456C>T			2 (2.5)
D-Loop	462C>T			5 (6.2)
D-Loop	468C>T			1 (1.2)
D-Loop	482T>C			3 (3.7)
D-Loop	487A>G			1 (1.2)
D-Loop	489T>C			9 (11.2)
D-Loop	497C>T		1 (9.1)	2 (2.5)
D-Loop	499G>A			1 (1.2)
D-Loop	523 delAC		1 (9.1)	5 (6.2)
**D-Loop**	**574A>C**			**2 (2.5)**
MT-RNR1	709G>A		3 (27.3)	10 (12.5)
MTRNR1	748G>A			1 (1.2)
MT-RNR1	750A>G		11 (100)	69 (86.2)

MTRNR1	930G>A			3 (3.7)
MTRNR1	961T>C			2 (2.5)
MTRNR1	980T>C			2 (2.5)
MTRNR1	1018G>A			1 (1.2)
MT-RNR1	1189T>C		1 (9.1)	4 (5.0)
MTRNR1	1243T>C			2 (2.5)
MT-RNR1	1438A>G		10 (90.9)	74 (92.5)
MTRNR1	1598G>A			2 (2.5)
MTRNR2	1700T>C			3 (3.7)
MTRNR2	1719G>A			10 (12.5)
MT-RNR2	1811A>G		1 (9.1)	12 (15.0)
MTRNR2	1888G>A			5 (6.2)
MTRNR2	2259C>T			1 (1.2)
MTRNR2	2294A>G			2 (2.5)
MTRNR2	2352T>C			1 (1.2)
MTRNR2	2416T>C			1 (1.2)
MTRNR2	2639C>T			2 (2.5)
MT-RNR2	2706A>G		1 (9.1)	41 (51.2)
MTRNR2	2789C>T			1 (1.2)
MTRNR2	3010G>A			11 (13.7)
MTRNR2	3027T>C			2 (2.5)
MT-RNR2	3197T>C		1 (9.1)	6 (7.5)
MTRNR2	3200T>C			1 (1.2)
MTRNR2	3202T>C			1 (1.2)
**MTRNR2**	**3213A>G**			**1 (1.2)**
MT-TL1	3290T>C			1 (1.2)
MT-ND1	3316G>A	A-T		1 (1.2)
MT-ND1	3394T>C	Y-H		1 (1.2)
MT-ND1	3396T>C	syn		1 (1.2)
MT-ND1	3421G>A	V-I		1 (1.2)
**MT-ND1**	**3456T>C**	**syn**		**1 (1.2)**
MT-ND1	3480A>G	syn	1 (9.1)	3 (3.7)
MT-ND1	3505A>G	T-A		2 (2.5)
MT-ND1	3546C>A	syn		2 (2.5)
MT-ND1	3579A>G	syn		13 (16.2)
MT-ND1	3594C>T	syn		1 (1.2)
MT-ND1	3834G>A	syn		1 (1.2)
MT-ND1	3921C>A	syn		2 (2.5)
**MT-ND1**	**3984C>T**	**syn**		**1 (1.2)**
MT-ND1	3992C>T	T-M	1 (9.1)	1 (1.2)
**MT-ND1**	**4002T>C**	**syn**		**1 (1.2)**
MT-ND1	4011C>T	syn		1 (1.2)
MT-ND1	4029C>T	syn		1 (1.2)
**MT-ND1**	**4050C>T**	**syn**		**1 (1.2)**
MT-ND1	4093A>G	T-A		2 (2.5)
MT-ND1	4104A>G	syn		1 (1.2)
MT-ND1	4188A>G	syn		3 (3.7)
MT-ND1	4216T>C	Y-H		13 (16.2)

**MT-ND1**	**4221C>A**	**syn**		**1 (1.2)**
MT-ND1	4232T>C	I-T		1 (1.2)
**MT-ND1**	**4243A>G**	**syn**		**1 (1.2)**
MT-TQ	4336T>C	T-C		1 (1.2)
**MT-ND2**	**4512G>A**	**A-T**		**1 (1.2)**
MT-ND2	4529A>T	syn		2 (2.5)
MT-ND2	4561T>C	V-A		1 (1.2)
MT-ND2	4580G>A	syn		2 (2.5)
MT-ND2	4586T>C	syn		1 (1.2)
MT-ND2	4639T>C	I-T		1 (1.2)
MT-ND2	4640C>A	I-M		3 (3.7)
MT-ND2	4646T>C	syn		2 (2.5)
MT-ND2	4659G>A	A-T		1 (1.2)
**MT-ND2**	**4677C>T**	**L-F**		**1 (1.2)**
MT-ND2	4688T>C	syn		1 (1.2)
MT-ND2	4703T>C	syn		2 (2.5)
MT-ND2	4736T>C	syn		1 (1.2)
**MT-ND2**	**4749T>C**	**S-P**		**1 (1.2)**
MT-ND2	4769A>G	syn	11 (100)	73 (91.2)
MT-ND2	4793A>G	syn		1 (1.2)
MT-ND2	4907T>C	syn		1 (1.2)
MT-ND2	4917A>G	N-D		6 (7.5)
**MT-ND2**	**4953A>G**	**I-V**		**1 (1.2)**
MT-ND2	4960C>T	A-V		2 (2.5)
MT-ND2	5004T>C	syn	1 (9.1)	1 (1.3)
MT-ND2	5046G>A	V-I		2 (2.5)
MT-ND2	5054G>A	syn		2 (2.5)
**MT-ND2**	**5081T>C**	**syn**		**1 (1.2)**
MT-ND2	5130T>C	syn		1 (1.2)
**MT-ND2**	**5135C>T**	**syn**		**1 (1.2)**
MT-ND2	5147G>A	syn	1 (9.1)	3 (3.7)
MT-ND2	5198A>G	syn		2 (2.5)
MT-ND2	5261G>A	syn		1 (1.2)
MT-ND2	5319A>G	T-S		2 (2.5)
MT-ND2	5360C>T	syn		1 (1.2)
MT-ND2	5460G>A	A-T		3 (3.7)
MT-ND2	5471G>A	syn	1 (9.1)	3 (3.7)
MT-ND2	5495T>C	syn		1 (1.2)
MT-ND2	5498A>G	syn		1 (1.2)
**MT-TW**	**5530C>T**			**1 (1.2)**
MT-TA	5633C>T			3 (3.7)
MT-TA	5655T>C			1 (1.2)
MT-NC4	5656A>G			1 (1.2)
MT-CO1	5999T>C	syn		1 (1.2)
MT-CO1	6026G>A	syn		1 (1.2)
MT-CO1	6047A>G	syn		1 (1.2)
MT-CO1	6125A>G	syn		1 (1.2)
**MT-CO1**	**6126A>G**	**I-V**		**1 (1.2)**

MT-CO1	6173C>T	syn		1 (1.2)
MT-CO1	6221T>C	syn	1 (9.1)	6 (7.5)
MT-CO1	6261G>A	A-T		1 (1.2)
MT-CO1	6266A>G	syn		1 (1.2)
**MT-CO1**	**6297T>C**	**syn**		**1 (1.2)**
**MT-CO1**	**6314C>A**	**syn**		**1 (1.2)**
MT-CO1	6359A>G	syn		1 (1.2)
MT-CO1	6365T>C	syn		1 (1.2)
MT-CO1	6371C>T	syn		5 (6.2)
**MT-CO1**	**6518C>T**	**syn**		**1 (1.2)**
MT-CO1	6524T>C	syn		2 (2.5)
MT-CO1	6546C>T	L-F		1 (1.2)
MT-CO1	6599A>G	syn		1 (1.2)
MT-CO1	6629A>G	syn		2 (2.5)
MT-CO1	6663A>G	syn		1 (1.2)
MT-CO1	6719T>C	syn		2 (2.5)
MT-CO1	6734G>A	syn		3 (3.7)
MT-CO1	6776T>C	syn	1 (9.1)	2 (2.5)
**MT-CO1**	**6899G>A**	**syn**		**1 (1.2)**
**MT-CO1**	**6975T>C**	**syn**		**1 (1.2)**
MT-CO1	7028C>T	syn	4 (36.4)	51 (63.7)
MT-CO1	7055A>G	syn		2 (2.5)
MT-CO1	7076A>G	syn		1 (1.2)
MT-CO1	7094T>C	syn		2 (2.5)
MT-CO1	7175T>C	syn		1 (1.2)
MT-CO1	7184A>G	syn		1 (1.2)
MT-CO1	7226G>A	syn		1 (1.2)
MT-CO1	7247C>T	syn		1 (1.2)
MT-CO1	7256C>T	syn		1 (1.2)
MT-CO1	7274C>T	syn		1 (1.2)
MT-CO1	7299A>G	M-V		1 (1.2)
MT-TS1	7476C>T			2 (2.5)
MT-TD	7521G>A			1 (1.2)
MT-TD	7547T>C			1 (1.2)
**MT-TD**	**7572T>C**			**1 (1.2)**
MT-CO2	7598G>A	A-T		1 (1.2)
MT-CO2	7621T>C	syn		1 (1.2)
MT-CO2	7645T>C	syn		2 (2.5)
**MT-CO2**	**7746A>G**	**N-S**		**1 (1.2)**
MT-CO2	7768A>G	syn		1 (1.2)
MT-CO2	7771A>G	syn		1 (1.2)
MT-CO2	7789G>A	syn		1 (1.2)
MT-CO2	7844A>G	T-A		1 (1.2)
MT-CO2	7858C>T	syn		1 (1.2)
MT-CO2	7961T>C	syn		1 (1.2)
MT-CO2	7963A>G	syn		1 (1.2)
MT-CO2	8137C>T	syn		1 (1.2)
MT-CO2	8152G>A	syn		3 (3.7)

MT-CO2	8206G>A	syn		1 (1.2)
MT-CO2	8222T>C	syn		1 (1.2)
MT-CO2	8251G>A	syn		7 (8.7)
MT-ATP8	8389A>G	syn		1 (1.2)
MT-ATP8	8393C>T	P-S		1 (1.2)
MT-ATP8	8448T>C	M-T		1 (1.2)
MT-ATP8	8460A>G	N-S		1 (1.2)
MT-ATP8	8472C>T	P-L		2 (2.5)
**MT-ATP8**	**8555T>C**	**syn**		**1 (1.2)**
MT-ATP6	8572G>A	G-S		1 (1.2)
MT-ATP8	8572G>A	Ter-Ter		1 (1.2)
**MT-ATP6**	**8596A>G**	**I-V**		**1 (1.2)**
MT-ATP6	8614T>C	syn		1 (1.2)
**MT-ATP6**	**8633A>G**	**Y-L**		**1 (1.2)**
MT-ATP6	8684C>T	T-I		1 (1.2)
MT-ATP6	8697G>A	syn		6 (7.5)
MT-ATP6	8701A>G	T-A		2 (2.5)
MT-ATP6	8705T>C	M-T		1 (1.2)
**MT-ATP6**	**8806C>T**	**P-S**		**1 (1.2)**
MT-ATP6	8818C>T	syn		1 (1.2)
MT-ATP6	8836A>G	M-V		2 (2.5)
MT-ATP6	8860A>G	T-A	10 (90.9)	69 (86.2)
MT-ATP6	8865G>A	syn		1 (1.2)
MT-ATP6	8869A>G	M-V		1 (1.2)
MT-ATP6	8938A>G	I-V		1 (1.2)
MT-ATP6	8994G>A	syn		1 (1.2)
MT-ATP6	9033A>G	syn		1 (1.2)
MT-ATP6	9090T>C	syn		4 (5.0)
MT-ATP6	9111T>C	syn		1 (1.2)
MT-ATP6	9123G>A	syn		1 (1.2)
**MT-ATP6**	**9139G>A**	**A-T**		**1 (1.2)**
**MT-CO3**	**9221A>G**	**syn**		**1 (1.2)**
**MT-CO3**	**9266G>A**	**syn**		**1 (1.2)**
**MT-CO3**	**9318C>T**	**H-Y**		**1 (1.2)**
MT-CO3	9335C>T	syn		2 (2.5)
MT-CO3	9452G>A	syn		1 (1.2)
MT-CO3	9477G>A	V-I	1 (9.1)	6 (7.5)
MT-CO3	9540T>C	syn		2 (2.5)
**MT-CO3**	**9656T>C**	**syn**		**4 (5.0)**
MT-CO3	9698T>C	syn	1 (9.1)	4 (5.0)
MT-CO3	9716T>C	syn		1 (1.2)
**MT-CO3**	**9742C>T**	**S-L**		**1 (1.2)**
MT-CO3	9756T>G	S-A		1 (1.2)
MT-CO3	9758T>C	syn		1 (1.2)
MT-CO3	9899T>C	syn		1 (1.2)
**MT-CO3**	**9903T>C**	**F-L**		**1 (1.2)**
MT-CO3	9932G>A	syn		2 (2.5)
MT-CO3	9948G>A	V-I		2 (2.5)

MT-CO3	9966G>A	V-I		2 (2.5)
MT-TG	10031T>C			1 (1.2)
MT-TG	10034T>C			2 (2.5)
MT-ND3	10115T>C	syn		1 (1.2)
MT-ND3	10142C>T	syn		1 (1.2)
MT-ND3	10143G>A	G-S		1 (1.2)
MT-ND3	10166T>C	syn		1 (1.2)
MT-ND3	10172G>A	syn		1 (1.2)
MT-ND3	10191T>C	S-P		1 (1.2)
MT-ND3	10217A>G	syn		3 (3.7)
MT-ND3	10237T>C	I-T		1 (1.2)
MT-ND3	10238T>C	syn		1 (1.2)
MT-ND3	10283A>G	syn		6 (7.5)
**MT-ND3**	**10322T>C**	**syn**		**1 (1.2)**
**MT-ND3**	**10334C>T**	**syn**		**1 (1.2)**
**MT-ND3**	**10356C>T**	**syn**		**1 (1.2)**
MT-ND3	10398A>G	T-A	1 (9.1)	13 (16.2)
MT-ND3	10400C>T	syn		1 (1.2)
MT-TR	10463T>C			7 (8.7)
MT-ND4L	10506A>G	T-A		2 (2.5)
MT-ND4L	10550A>G	syn	1 (9.1)	3 (3.7)
MT-ND4L	10586G>A	syn		1 (1.2)
**MT-ND4L**	**10739A>G**	**syn**		**1 (1.2)**
MT-ND4	10810T>C	syn		2 (2.5)
MT-ND4	10873T>C	syn		2 (2.5)
MT-ND4	10978A>G	syn		1 (1.2)
MT-ND4	11016G>A	S-N		1 (1.2)
MT-ND4	11025T>C	C-P		1 (1.2)
MT-ND4	11061C>T	S-F		1 (1.2)
MT-ND4	11242C>G	syn		1 (1.2)
MT-ND4	11251A>G	syn		13 (16.2)
MT-ND4	11299T>C	syn	1 (9.1)	4 (5.0)
MT-ND4	11332C>T	syn		1 (1.2)
MT-ND4	11362A>G	syn		2 (2.5)
MT-ND4	11437T>C	syn		1 (1.2)
MT-ND4	11440G>A	syn		1 (1.2)
MT-ND4	11467A>G	syn	2 (18.2)	18 (22.5)
MT-ND4	11653A>G	syn		1 (1.2)
MT-ND4	11674C>T	syn		2 (2.5)
MT-ND4	11719G>A	syn	2 (18.2)	45 (56.2)
MT-ND4	11812A>G	syn		5 (6.2)
**MT-ND4**	**11827T>C**	**syn**		**1 (1.2)**
MT-ND4	11914G>A	syn		3 (3.7)
MT-ND4	11944T>C	syn		1 (1.2)
MT-ND4	11947A>G	syn		2 (2.5)
MT-ND4	11971C>T	syn		1 (1.2)
MT-TS2	12234A>G			1 (1.2)
**MT-TL2**	**12246C>T**			**1 (1.2)**

MT-ND5	12346C>T	H-Y		2 (2.5)
MT-TL2	12308A>G		2 (18.2)	18 (22.5)
MT-ND5	12372G>A	syn	2 (18.2)	18 (22.5)
**MT-ND5**	**12378C>T**	**syn**		**1 (1.2)**
MT-ND5	12414T>C	syn		2 (2.5)
MT-ND5	12501G>A	syn		5 (6.2)
MT-ND5	12612A>G	syn		6 (7.5)
MT-ND5	12633C>T	syn		2 (2.5)
MT-ND5	12693A>G	syn		1 (1.2)
MT-ND5	12705C>T	syn		14 (17.5)
MT-ND5	12771G>A	syn		1 (1.2)
MT-ND5	12930A>G	syn		1 (1.2)
MT-ND5	12937A>G	M-V		1 (1.2)
MT-ND5	13368G>A	syn		6 (7.5)
MT-ND5	13617T>C	syn	1 (9.1)	8 (10.0)
MT-ND5	13708G>A	A-T		8 (10.0)
MT-ND5	13743T>C	syn		4 (5.0)
MT-ND5	13759G>A	A-T		2 (2.5)
MT-ND5	13780A>G	I-V		2 (2.5)
MT-ND5	13934C>T	T-M		3 (3.7)
MT-ND5	13966A>G	T-A		5 (6.2)
MT-ND5	14139A>G	syn		6 (7.5)
MT-ND6	14167C>T	syn	1 (9.1)	5 (6.2)
MT-ND6	14233A>G	syn		5 (6.2)
MT-ND6	14470T>C	syn		5 (6.2)
MT-ND6	14569G>A	syn	3 (27.3)	1 (1.2)
MT-CYB	14766C>T	T-I	2 (18.2)	45 (56.2)
MT-CYB	14769A>G	N-S		1 (1.2)
MT-CYB	14783T>C	syn		1 (1.2)
MT-CYB	14793A>G	H-R	1 (9.1)	5 (6.2)
MT-CYB	14798T>C	F-L	1 (9.1)	8 (10.0)
MT-CYB	14905G>A	syn		6 (7.5)
MT-CYB	15043G>A	syn		3 (3.7)
MT-CYB	15071T>C	Y-N		1 (1.2)
MT-CYB	15110G>A	A-T	1 (9.1)	1 (1.2)
MT-CYB	15218A>G	T-A		5 (6.2)
MT-CYB	15301G>A	syn		2 (2.5)
MT-CYB	15326A>G	T-A	11 (100)	71 (88.7)
MT-CYB	15452C>A	L-I		13 (16.2)
MT-CYB	15454T>C	syn		4 (5.0)
MT-CYB	15607A>G	syn		6 (7.5)
MT-TT	15904C>T			2 (2.5)
MT-TT	15910C>T			1 (1.2)
MT-TT	15924A>G			5 (6.2)
MT-TT	15928G>A			6 (7.5)
D-Loop	16069C>T			7 (8.7)
D-Loop	16074A>G			1 (1.2)
D-Loop	16086T>C			1 (1.2)

D-Loop	16093T>C		4 (5.0)
D-Loop	16126T>C		15 (18.7)
D-Loop	16129G>A	1 (9.1)	3 (3.8)
D-Loop	16134C>T		1 (1.2)
D-Loop	16140T>C		2 (2.5)
D-Loop	16142C>T		1 (1.2)
D-Loop	16145G>A		2 (2.5)
D-Loop	16148C>T		1 (1.2)
D-Loop	16150C>T		1 (1.2)
D-Loop	16153G>A		1 (1.2)
D-Loop	16154T>C		1 (1.2)
D-Loop	16162A>G		1 (1.2)
D-Loop	16163A>G		1 (1.2)
D-Loop	16172T>C		1 (1.2)
D-Loop	16176C>G		2 (2.5)
D-Loop	16180A>G		1 (1.2)
D-Loop	16182A>C		1 (1.2)
D-Loop	16183A>C		4 (5.0)
D-Loop	16184C>T		1 (1.2)
D-Loop	16185C>T		1 (1.2)
D-Loop	16186C>T		1 (1.2)
D-Loop	16188C>T	3 (27.3)	1 (1.2)
D-Loop	16189T>C	1 (9.1)	12 (15.0)
D-Loop	16192C>T	1 (9.1)	7 (8.8)
D-Loop	16193C>T		4 (5.0)
D-Loop	16224T>C	1 (9.1)	5 (6.2)
D-Loop	16233A>G	1 (9.1)	13 (16.2)
D-Loop	16256C>T	1 (9.1)	9 (11.2)
D-Loop	16260C>T		2 (2.5)
D-Loop	16270C>T	1 (9.1)	9 (11.2)
D-Loop	16278C>T	1 (9.1) [a]	6 (7.5)
**D-Loop**	**16285A>C**		**1 (1.2)**
D-Loop	16286C>A		1 (1.2)
D-Loop	16288T>C		3 (3.7)
D-Loop	16289A>G		5 (6.2)
D-Loop	16290C>A		1 (1.2)
D-Loop	16292C>T		3 (3.7)
D-Loop	16293A>C		9 (11.2)
D-Loop	16294C>T		7 (8.7)
D-Loop	16296C>T		4 (5.0)
D-Loop	16298T>C	2 (18.2)	3 (3.7)
D-Loop	16304T>C		7 (8.7)
D-Loop	16311T>C	3 (27.3)	13 (16.2)
D-Loop	16325T>C		3 (3.7)
D-Loop	16328C>A		4 (5.0)
D-Loop	16352T>C		4 (5.0)
D-Loop	16355C>T		2 (2.5)
D-Loop	16362T>C	1 (9.1)	4 (5.0)

D-Loop	16384G>A		6 (7.5)
D-Loop	16390G>A		6 (7.5)
D-Loop	16391G>A		2 (2.5)
**D-Loop**	**16402A>C**		**2 (2.5)**
D-Loop	16519T>C	7 (63.6)	29 (36.2)
D-Loop	16527C>T		1 (1.2)

In bold are reported novel variants; [a]Variant present at heteroplasmic level.

For previously described variants see www.mitomap.org.

The variants detected in Mitochondrial diabetic affected patients were always present also in their mothers.
